# Molecular Mechanism of Curcumin Derivative on YAP Pathway against Ovarian Cancer

**DOI:** 10.3390/jcm11237220

**Published:** 2022-12-05

**Authors:** Nan Zheng, Shan Liu, Huiting Zeng, Huajun Zhao, Lixu Jin

**Affiliations:** 1 School of Pharmacy, Zhejiang Chinese Medical University, Hangzhou 310053, China; 2 School of Pharmacy, Wenzhou Medical University, Wenzhou 325015, China; 3 Obstetric Department, First Affiliated Hospital of Wenzhou Medical University, Wenzhou 325015, China

**Keywords:** ovarian cancer, curcumin, WZ10, apoptosis, Hippo-YAP pathway, apoptosis

## Abstract

The purpose of this study is to study the effect of curcumin derivative WZ10 on the proliferation, invasion and apoptosis of ovarian cancer OVCAR3 cells, and to explore its mechanism. MTT assay was used to detect the effect of WZ10 on the proliferation of ovarian cancer OVCAR3 cells; Annexin V/PI double staining flow cytometry was used to detect the effect of WZ10 on cell apoptosis; Transwell method was used to detect the effect of WZ10 on cell invasiveness; Western blot was used to investigate the effect of WZ10 Mechanisms affecting OVCAR3 activity in ovarian cancer in vitro. Our results show that WZ10 treatment could significantly inhibit the proliferation and invasion of OVCAR3 cells, induce apoptosis of OVCAR3 cells in a dose-dependent manner. After knockdown of Hippo expression with sh-RNA, further combined treatment with WZ10 had no significant image on ovarian cancer OVCAR3 cells. In conclusion: WZ10 can significantly inhibit the proliferation of OVCAR3 cells, reduce cell invasion and proliferation by downregulating the activation of Hippo-YAP pathway, and induce cell apoptosis.

## 1. Introduction

Ovarian cancer, a common malignant tumor of the female reproductive system, is the most fatal of all female malignant tumors [1]. Platinum is the primary medication for chemotherapy of ovarian cancer. However, platinum resistance and serious adverse reactions have become difficult clinical problems, and the development of new antitumor drugs is a hot spot in current ovarian cancer treatment research [2,3]. Traditionally, screening of small molecule inhibitors of a target requires a large amount of money to obtain the physical drug and then perform enzymatic-level screening one by one, followed by in vitro validation. This drug screening process is time-consuming, costly and inefficient. As a result, more and more research institutions are adopting “artificial intelligence” drug design screening systems, which will be based on machine learning techniques to simulate the drug properties of small molecule compounds and thus select the most promising mock compounds for synthesis and experimentation. Curcumin-derived WZ10 was used as a drug candidate for this study.

Curcumin (Curcumin) is a polyphenolic substance extracted from the genus Zingiberaceae, with antimutation, antioxidant and other biological properties [4]. It has a certain inhibitory effect on malignant tumors of many systems, but the bioavailability of curcumin is poor, and its clinical efficacy is reduced [4,5,6]. A CUR analog WZ10, obtained by our research group through molecular structure modification on the basis of retaining the activity of CUR, has higher stability and bioavailability, and previous research has discovered that it has a good proliferation inhibitory effect on ovarian cancer cells OVCAR3, with an IC50 of 2.9 μM.

With the progress of functional studies, there is evidence that numerous signaling pathways contribute to the development of tumors [7,8]. The Hippo-YES-associated protein (YAP) signaling pathway promotes cell proliferation, inhibits apoptosis and promotes the growth of stem (progenitor) cells in epithelial tissues, which is crucial for controlling organ size [9,10,11]. Antibody binding to the amino-terminal region of the Yes protein allowed for the initial identification of YAP. Hippo pathway kinase negatively regulates YAP protein, which leads to YAP binding to 14-3-3 proteins through phosphorylation of YAP serine 127, and eventually causes cytoplasmic arrest and degradation of YAP [3]. YAP is a transcription coactivator and a significant downstream impactor molecule of the Hippo pathway, which binds with transcription factors TEADs in the nucleus. Transcription of downstream genes is initiated to promote proliferation and inhibit apoptosis [4]. YAP is often expressed at high levels in a wide range of human solid tumors, such as liver cancer, gastric cancer and colorectal cancer [11,12,13]. In order to evaluate WZ10’s effects on ovarian cancer OVCAR3 cells’ invasion, spreading and apoptosis in vitro, and to learn more about how it works, the ovarian cancer OVCAR3 cell line sensitive to cisplatin and doxorubicin was chosen as the model.

## 2. Methods and Reagents

### 2.1. Reagents

The Chinese Academy of Sciences’ Institute of Biochemistry and Cell Biology supplied the ovarian cancer cell line that was purchased. WZ10 was purchased from Sigma (St. Louis, MO, USA). Phospho-YAP (Ser127) (D9W2I) Rabbit mAb (#13008), MST1 (D8B9Q) Rabbit mAb (#14946), MST2 Antibody (#3952), LATS1 (C66B5) Rabbit mAb (#3477), Phospho-MOB1 (Thr12) (D2E3) Rabbit (mAb #8843), β-Actin (8H10D10) Mouse mAb (#3700), Cyclin D1 (E3P5S) XP^®^ Rabbit mAb (#55506), Oct-4A (C52G3) Rabbit mAb (#2890) and Vimentin (D21H3) XP^®^ Rabbit mAb (BSA and Azide Free) (#46173) were purchased from Cell Signaling Technology (Boston, MA, USA). The immunohistochemistry kit was purchased from Beijing Zhongshan Jinqiao Biotechnology Co., LTD. Fetal bovine serum, antibiotics, DMEM medium (Gibco) and 5-Bromo-2-deoxyUridine (BrdU) cell proliferation assay kit were purchased from LifeTechnologies (Gaithersburg, MD, USA). YAP shRNA and the transfection kit Effectene^R^ Transfection Reagent were purchased from QIAGEN, USA. Annexin V-FLUOS Apoptosis Detection Kit was purchased from Roche (Basel, Switzerland). FACScalibur flow cytometer was purchased from Becton-Dickinson (Franklin Lake, NJ, USA). HybridMulti-Mode Microplate Readers Microplate Reader was purchased from Omega Biotek (Norcross, GA, USA).

### 2.2. Cell Culture

In a cell culture incubator with 5% CO_2_ at 37 °C and saturated humidity, the cells were cultured in DMEM medium containing 100 mL/L fetal bovine serum, 100 U/mL penicillin and 100 g/mL streptomycin. After stable passage for 2–3 passages, cells in logarithmic growth phase were taken for experiments.

### 2.3. siRNA Transfection

Purified Hippo shRNA (experimental group) and NT shRNA (control group) were placed in the transfection reagent package according to the instructions for Effectene^R^ Transfection Reagent. The old culture medium was discarded, 3 mL fresh culture medium and 1 mL disease venom were added, and then 8 μg/mL polybrene was added. The cell incubator was incubated overnight, and the fresh DMEM medium was replaced on the second day, and the virus was infected for 48–96 h.

### 2.4. Cell Grouping and MTT

Six-well plates were used to seed OVCAR3 cells, and cells were divided into control group (PBS treated), WZ10 group (including low-dose, medium-dose and high-dose groups and used 1, 2 or 4 µM WZ10, respectively, for treatment), sh-NC group (transfected with nonsense siRNA), si-Hippo group (transfected with Hippo siRNA) and si-Hippo + WZ group (treated with 4 µM WZ10 after Hippo siRNA transfection).

After 24 h of different treatments, according to the instructions from the reagent manufacturer, each well received 10μl of MTT reagent which was added, and after 2 h of incubation at room temperature, the absorbance was determined at 450 nm.

### 2.5. Flow Cytometry

Trypsin-digested cells were collected in accordance with the kit’s instructions, and (1–5) 10^5^ cell suspension solution along with 500 mL of binding buffer, 5 mL of Annexin V-FITC and 10 mL of propidium iodide were then added (PI). The test was completed in 1 h, after the reaction was kept out of light for 5 to 15 min at room temperature.

### 2.6. Transwell

Prior to the invasion test, matric gel was used to humidify the Transwell (the chamber was filled with 40 μL of serum-free medium, and the cells were incubated there for two hours at 37 °C). By digesting them with trypsin, we gathered SKOV3 cells in the logarithmic growth phase. The cells were suspended in RPMI-1640 medium containing low toxic concentrations of TP-PEI-CyD and TPL. The cells were diluted to 5 × 106 cells per mL, and the cell suspension was added to a Transwell chamber with 3 wells per chamber and 3 parallel wells. The chamber was placed in a 24-well plate and cultured for 24 h at 37 ℃ in a 5% CO_2_ incubator. In the lower chamber, 600 μL of DMEM medium containing 10% serum was meticulously added, and the culture was continued for 24 h. The chamber was then removed, fixed with formaldehyde for five minutes, the proper amount of Giemsa application solution added, and stained for ten to thirty minutes, and then a cotton swab was used to gently wipe off the cells that had not made it through the membrane. An inverted microscope was used to choose five high-power fields, and it was counted how many cells made it through the bottom membrane.

### 2.7. Western Blot

After removing the treated cells and pouring out the DMEM medium, PBS was used to thoroughly rinse the cells. RIPA cell lysate was then used to extract the total protein, and its concentration was assessed using the BCA method. The gel was electrophoresed at 110 V for 1.5 h, wet at 110 V at 4 °C for 1.5 h and coagulated with 12% polyacrylamide. After being blocked at room temperature for 10 min with 5% defatted milk powder (dissolved with TBST), the YAP antibody was added and incubated at 4 °C overnight. TBST was rinsed for 5 min × 3 times, then donkey anti-rabbit secondary antibody was added, diluted 1:1000 and incubated for 2 h at room temperature. It was then rinsed with TBST for 5 min × 3 times. ECL liquid was immersed in the dark room to develop color, and the film was exposed. The automatic X-ray film washer was used to clean the film.

### 2.8. RT-PCR

After collection, total RNA was taken from each group of cells. The cDNA was stored at −20 °C for later use after the reverse transcription reaction was completed in keeping with the kit’s procedure. The following were the reaction conditions: predenaturation at 94 °C for 5 min, treatment at 95 °C for 5 s and reaction at 60 °C for 30 s were carried out for 45 cycles. Three independent measurements were performed for each group. To determine the relative mRNA content, an image analyzer scanned the gel density. Table 1 lists the PCR primers that were used to amplify the indicated genes. The samples’ relative expression was normalized to that of the internal control, GAPDH.

### 2.9. Statistical Analysis

SPSS 22.0 was used to conduct the statistical analysis. Data from measurements were presented as mean standard deviation (x ± s). ANOVA was used to compare groups in one way, the t-test to compare two groups and the χ^2^ test to compare count data. When *p* < 0.05, it was regarded as statistically significant.

## 3. Results

### 3.1. WZ10 Promotes Apoptosis and Reduces Ovarian Cancer Cell Invasion and Proliferation

**Figure 1:**
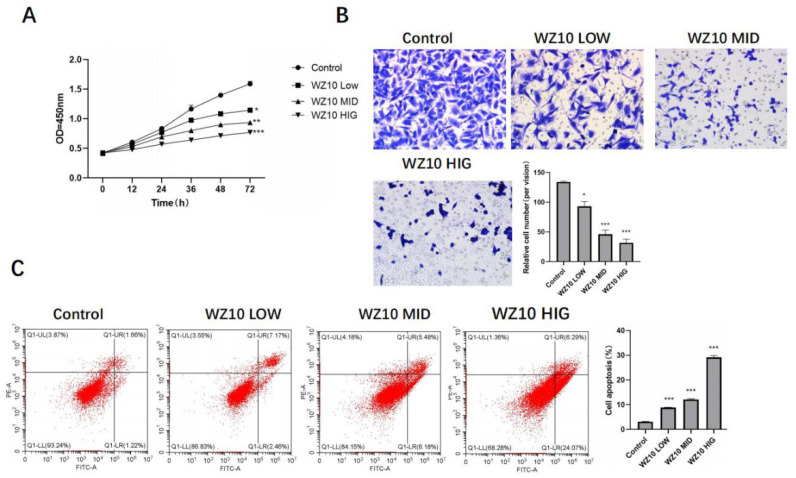
In vitro anti-ovarian cancer activity of WZ10. After treatment with different concentrations of WZ10: (**A**) detection of ovarian cancer cell proliferation activity by MTT; (**B**) detection of ovarian cancer cell invasion activity by Transwell, original image × 50; (**C**) detection of ovarian cancer cell apoptosis level by flow cytometry. Compared with control group, * *p* < 0.05, ** *p* < 0.01, *** *p* < 0.001.

After treatment with different concentrations of WZ10, the ability of ovarian cancer cells to proliferate was significantly diminished. Transwell experiments indicated that WZ10 could significantly reduce ovarian cancer cells’ ability to invade, and flow apoptosis experiments indicated that WZ10 could significantly promote the apoptosis of ovarian cancer cells and showed a dose-dependent manner (Figure 1A–C).

### 3.2. WZ10 Inhibits Activation of the Hippo-YAP Pathway

**Figure 2:**
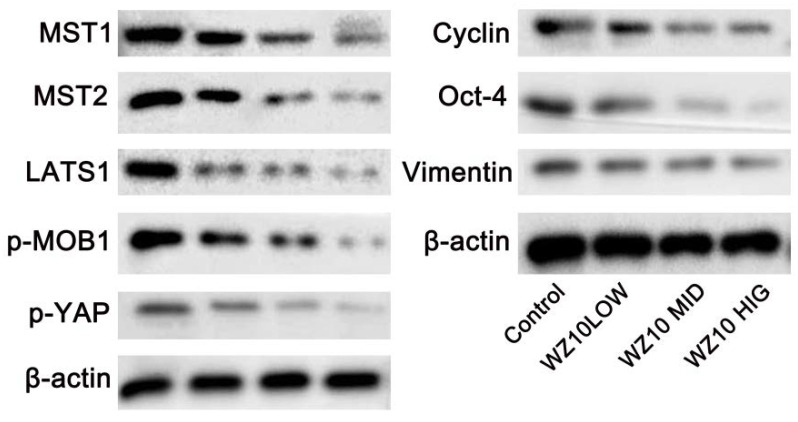
WZ10 inhibits Hippo-YAP pathway in ovarian cancer cells.

In ovarian cancer cells treated with WZ10, Western blotting revealed that the expression levels of Hippo-YAP pathway-related proteins MST1/2, LATS1, p-MOB1 and p-YAP were markedly lower than in control cells. The expressions of YAP downstream effectors cyclin, Oct-4 and vimentin were also significantly downregulated in a dose-dependent manner (Figure 2).

### 3.3. In Vitro, Hippo Knockdown Reduces the Activity of Ovarian Cancer

**Figure 3:**
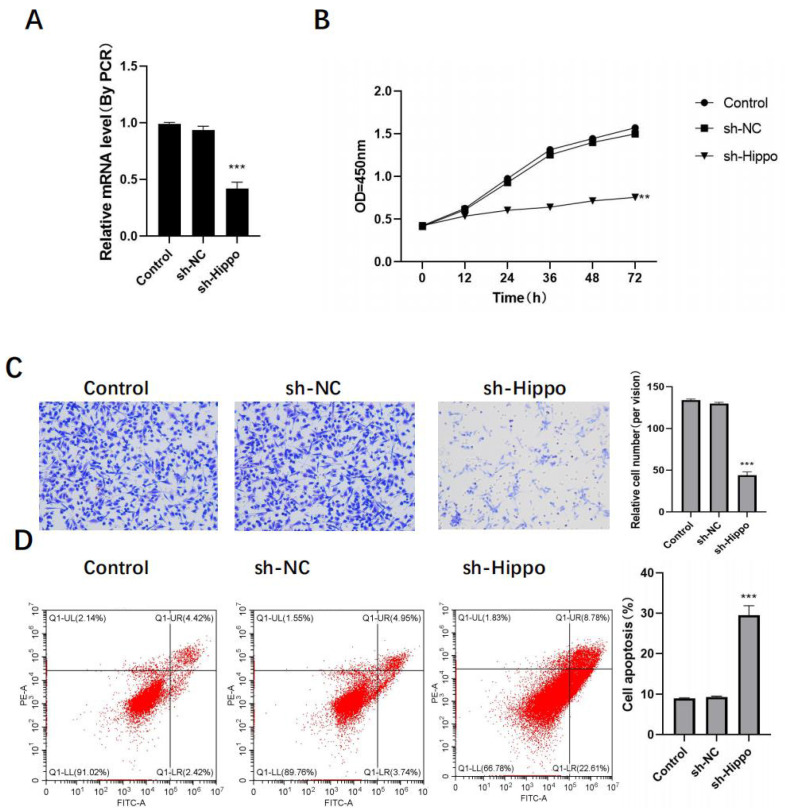
Knockdown of Hippo inhibits the activity of ovarian cancer cells in vitro. After Hippo transfection: (**A**) sh-Hippo transfection efficiency was detected by RT-PCR; (**B**) detection of ovarian cancer cell proliferation activity by MTT; (**C**) detection of ovarian cancer cell invasion activity by Transwell, original image × 50; (**D**) detection of ovarian cancer cell apoptosis level by flow cytometry. Compared with control group, ** *p* < 0.01, *** *p* < 0.001.

OVCAR3 cells were transfected using sh-Hippo, and RT-PCR analysis revealed that, in comparison to control cells, the expression of Hippo in the sh-Hippo group was significantly reduced (Figure 3A). Additionally, ovarian cancer cells’ proliferation and metastasis were also significantly reduced, while their level of apoptosis was significantly increased (Figure 3B–D).

### 3.4. WZ10 Exerts Anticancer Activity through the Hippo-YAP Pathway

**Figure 4:**
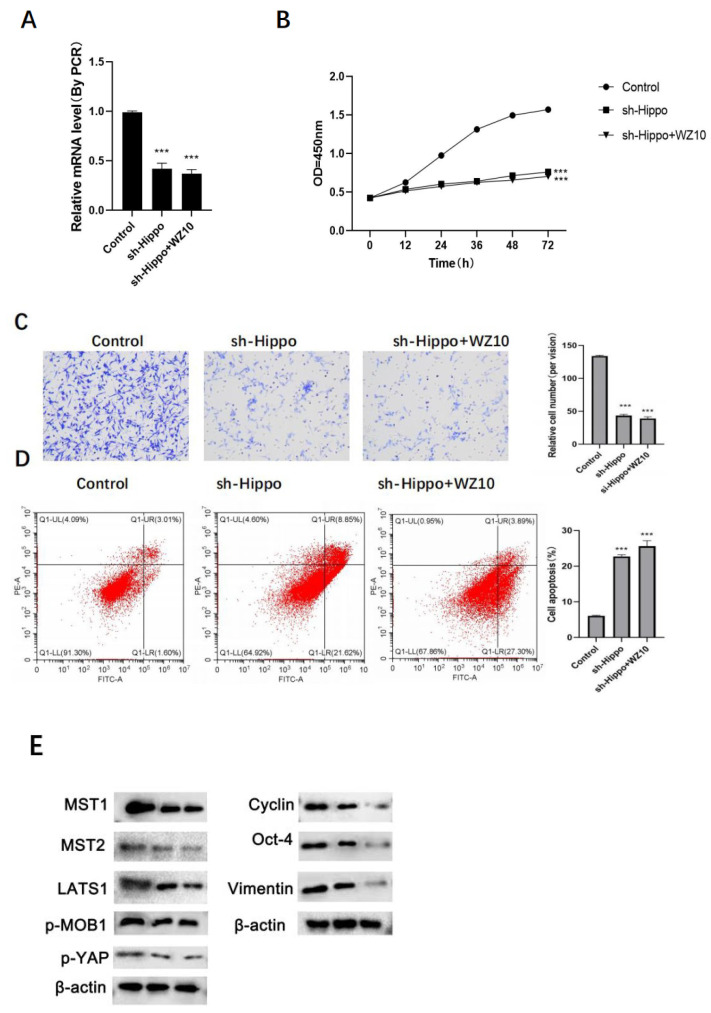
Mechanism of WZ10 inhibiting the activity of ovarian cancer cells. After different treatments: (**A**) the expression of Hippo was detected by RT-PCR; (**B**) detection of ovarian cancer cell proliferation activity by MTT; (**C**) detection of ovarian cancer cell invasion activity by Transwell, original image × 50; (**D**) detection of ovarian cancer cell apoptosis level by flow cytometry. Compared with control group, (E)Protein expression levels after different treatments,*** *p* < 0.001.

In vitro experiments showed that compared with the sh-Hippo group cells, the expression of Hippo in ovarian cancer cells, the proliferation ability, metastasis ability and apoptosis level of ovarian cancer cells did not show significant change after the combined treatment of sh-Hippo and WZ10 (Figure 4A–D).

## 4. Discussion

In our previous study, we conducted a natural product small molecule screening for ovarian cancer and proposed to use an “artificial intelligence” drug screening to obtain the best candidate small molecule, curcumin-derived WZ10, from thousands of natural products, and then use this small molecule for follow-up studies. In recent years, the antitumor effect of CUR has attracted attention. Some studies have found that CUR can prevent tumor angiogenesis and cell proliferation, induce cancer cell apoptosis, downregulate epidermal growth factor receptor (EGFR) activity and human epidermal growth factor receptor 2 (HER2) expression levels, downregulate nuclear transcription factor, inhibit the activation of the STAT3 signaling pathway, downregulate the expression level of cyclooxygenase 2 (COX-2), and reduce the synthesis level of MMP9 and inducible nitric oxide synthase (iNOS), thus exerting its antitumor effect through multiple pathways [14,15]. However, the application of CUR is constrained by its low water solubility, ease of aqueous solution hydrolysis, clear first-pass elimination effect and low bioavailability [16]. Obtaining CUR analogs or derivatives through molecular structure modification can well improve the bioavailability of drugs [17]. CUR is a symmetric double carbonyl structure, which is unstable. The School of Pharmacy, Wenzhou Medical University, obtained a series of CUR analogs through molecular structure modification, screened their antitumor activities and selected compounds with better stability and antitumor activity for further research, of which WZ10 is one. In this investigation, it was discovered that WZ10 dramatically inhibited OVCAR3 cells, and its inhibitory activity was significantly better than its parental CUR.

As a chemical monomer extracted from Zingiberaceae, curcumin has a wide range of biological effects. Wang Jian et al. [4] found that curcumin could exert anti-inflammatory effects by inhibiting inflammatory mediators and transcription factors such as cyclooxygenase-2 (COX-2) and nuclear factor-kappa B (NF-κB). Curcumin is regarded as a potent antioxidant as well. Studies have shown that it can withstand oxidative stress by neutralizing free radicals, triggering antioxidant enzymes such as catalase and superoxide dismutase and preventing lipid peroxidation [5,6]. In addition, curcumin can exert antitumor effects through various mechanisms [7]. The most prevalent subtype of ovarian cancer and the primary reason for cancer-related deaths in women worldwide [1], around 2.1 million new cases and more than 626,000 deaths from ovarian cancer were reported in 2018 [2].

Ovarian cancer cells often have strong proliferative and invasive abilities, which is also an important reason why ovarian cancer is difficult to cure. This study used the curcumin derivative WZ10 to treat ovarian cancer cells. The results revealed that WZ10 treatment could significantly and dose-dependently inhibit ovarian cancer’s ability to proliferate and invade. In order to maintain cell homeostasis, Hippo-YAP signaling can play a role in the control of physiological processes such as cell differentiation, proliferation and apoptosis. It is a comparatively conservative signaling pathway that is crucial for the emergence and growth of a variety of tumors, including ovarian cancer [9,10,11]. The WZ10 treatment in the current study significantly decreased the protein levels of important Hippo pathway constituents, including MST1/2, LATS1, phosphorylated MOB1 and p-YAP, indicating that the Hippo pathway was not activated.

The Hippo-YAP/TAZ pathway is mainly composed of upstream regulatory molecules, intermediate core kinases and downstream transcription factors [2]. In mammals, the Hippo-YAP/TAZ pathway is composed of a large protein network. Its core pathway is an important tumor suppressor, which is composed of mammalian sterile 20-like kinase 1/2, MST1/2, salvador homologue 1(SAV1) containing WW domain, large tumor suppressor 1/2 (SAV1), LATS1/2) and 1 MOB kinase activator (MOB kinase activator 1, MOB1) A. MOB1B constitutes the core of the kinase box, and when the Hippo-YAP/TAZ pathway is activated, MST1/2, with the help of SAV1, Phosphorylated MOB1 and LATS1/2, enhances the auxiliary activation of MOB1 on LATS1/2. Phosphorylated LATS1/2 can inactivate the transcriptional coactivator YAP/TAZ by phosphorylation at multiple sites after activation, so that the latter can be retained in the cytoplasm and degraded by ubiquitination. Thus, the expression of downstream target genes is hindered, cell proliferation and apoptosis are controlled and tumorigenesis is inhibited [4]. Key nuclear effector molecules downstream of the Hippo-YAP/TAZ pathway, YAP and TAZ, are homologous proteins that are inhibited by the activated Hippo-YAP/TAZ pathway [5]. When Hippo signaling is turned off, YAP/TAZ is activated and enters the nucleus, where it can bind to TEA domain family members (TEAD) and promote transforming growth factors, interleukins (leukins), IL) -6, dimodulin, cyclin E and a series of genes related to cell proliferation, thereby promoting tumorigenesis [5]. The Hippo-YAP/TAZ channel controls YAP and TAZ through a series of upstream machines, and YAP and TAZ are the keys to regulate and output the Hippo-YAP/TAZ channel. The abnormal regulation of the Hippo-YAP/TAZ pathway induces abnormal activation of YAP/TAZ, which leads to tumorigenesis and endows cancer stem cells with characteristics such as promoting cell proliferation, inhibiting cell apoptosis and causing changes in the tumor immune microenvironment.

LATS1 can phosphorylate YAP, a significant Hippo pathway downstream effector, thereby inhibiting YAP nuclear translocation [18,19]. Together with TEAD, YAP functions in the nucleus as a transcriptional coactivator to suppress RB/p16 expression and promote the expression of cyclin, Oct-4 and vimentin, which are connected to cell proliferation, cell cycle arrest and cellular senescence [20,21,22]. After ovarian cancer cells were treated with WZ10, there was a dose-dependent, significant downregulation in the expressions of cyclin, Oct-4 and vimentin, suggesting that WZ10 treatment could inhibit the activation of the Hippo-YAP pathway. In a similar manner, Western blot analysis confirmed that using sh-RNA to knock down Hippo can significantly prevent the activation of Hippo-YAP signaling, thereby inhibiting the in vitro activity of ovarian cancer. This inhibition is manifested as a decrease in the proliferation and invasion ability of ovarian cancer cells, as well as a significant increase in the level of apoptosis.

Tumor cytoreductive surgery or chemotherapy drugs can significantly improve the majority of advanced ovarian cancer patients, but it is simple to relapse, and once relapse occurs, the prognosis is very poor; the 5-year survival rate of advanced ovarian cancer patients is only 20%–30%. Therefore, it is crucial to increase the number of ovarian cancer patients who receive an early diagnosis and to research more efficient treatments. Tumor therapy that targets the Hippo-YAP signaling pathway has gained popularity in recent years. YAP-like peptides created by Zhou et al. [14] have demonstrated their ability to inhibit tumor growth in primary liver cancer xenograft models. Our study indicates that WZ10 may decrease tumor activity in vitro through the Hippo-YAP pathway, induce cell apoptosis and limit cell proliferation and invasion, supporting WZ10 as a promising medication for the therapeutic treatment of ovarian cancer patients.

## Figures and Tables

**Table 1:** PCR primer sequences used.

Gen	Upstream Primer 5′-3′	Downstream Primer 5′-3′
Hippo	ATAAGTGCATCCCACACCCG-3	GCGTGCTCAGCAATTTCACA
GAPDH	ACAGTCAGCCGCATCTTCT	GACAAGCTTCCCGTTCTCAG
